# Barrier-Protective Effects of Activated Protein C in Human Alveolar Epithelial Cells

**DOI:** 10.1371/journal.pone.0056965

**Published:** 2013-02-22

**Authors:** Ferranda Puig, Gemma Fuster, Mélanie Adda, Lluís Blanch, Ramon Farre, Daniel Navajas, Antonio Artigas

**Affiliations:** 1 CIBER de Enfermedades Respiratorias, Bunyola, Spain; 2 Critical Care Center, Hospital de Sabadell, Institut Universitari Parc Taulí, Universitat Autònoma de Barcelona, Sabadell, Spain; 3 Unitat de Biofísica i Bioenginyeria, Facultat de Medicina, Universitat de Barcelona, Barcelona, Spain; 4 Institut d’Investigacions Biomèdiques August Pi i Sunyer (IDIBAPS), Barcelona, Spain; 5 Institut de Bioenginyeria de Catalunya (IBEC), Barcelona, Spain; University of Washington, United States of America

## Abstract

Acute lung injury (ALI) is a clinical manifestation of respiratory failure, caused by lung inflammation and the disruption of the alveolar-capillary barrier. Preservation of the physical integrity of the alveolar epithelial monolayer is of critical importance to prevent alveolar edema. Barrier integrity depends largely on the balance between physical forces on cell-cell and cell-matrix contacts, and this balance might be affected by alterations in the coagulation cascade in patients with ALI. We aimed to study the effects of activated protein C (APC) on mechanical tension and barrier integrity in human alveolar epithelial cells (A549) exposed to thrombin. Cells were pretreated for 3 h with APC (50 µg/ml) or vehicle (control). Subsequently, thrombin (50 nM) or medium was added to the cell culture. APC significantly reduced thrombin-induced cell monolayer permeability, cell stiffening, and cell contraction, measured by electrical impedance, optical magnetic twisting cytometry, and traction microscopy, respectively, suggesting a barrier-protective response. The dynamics of the barrier integrity was also assessed by western blotting and immunofluorescence analysis of the tight junction ZO-1. Thrombin resulted in more elongated ZO-1 aggregates at cell-cell interface areas and induced an increase in ZO-1 membrane protein content. APC attenuated the length of these ZO-1 aggregates and reduced the ZO-1 membrane protein levels induced by thrombin. In conclusion, pretreatment with APC reduced the disruption of barrier integrity induced by thrombin, thus contributing to alveolar epithelial barrier protection.

## Introduction

The pathogenesis of acute lung injury (ALI) and acute respiratory distress syndrome (ARDS) involves pro-coagulant and pro-inflammatory mechanisms resulting in disrupted alveolar epithelium at cell-cell junctions, with the consequent infiltration of protein-rich edema fluid and inflammatory cells into the alveolar space [1], [2]. Therefore, the physical integrity of the alveolar epithelial barrier plays an important role in these respiratory diseases. The integrity of this barrier depends on a dynamic balance between inward forces–cell mechanical tension generated by actomyosin contraction and cytoskeleton elastic recoil–and outward forces exerted by cell-cell and cell-matrix adhesions [3]. During the early stages of ALI/ARDS, pro-inflammatory mediators downregulate the natural anticoagulant pathways and initiate an increase in pro-coagulant activity [4]–[6], which could compromise the force balance in the alveolar epithelium. The serine protease thrombin is one of the most important pro-coagulant proteins that increase in the injured lungs of patients with clinical disorders resulting in ALI/ARDS [7]. Thrombin can modulate the force balance in alveolar epithelial cells by increasing cell stiffness [8] and cell contraction [9] and also by enhancing peripheral remodeling of the actin cytoskeleton [8]–[10] and of cell-cell contacts, such as the tight junction ZO-1 [10].

Activated protein C (APC) is an anticoagulant protein that is formed after the activation of protein C by thrombin-thrombomodulin complex on the surface of cells [11], including alveolar epithelial cells [5]. The endothelial protein C receptor (EPCR) accelerates protein C activation in a concentration-dependent manner. In ALI/ARDS patients, the generation of APC in alveolar compartments could be drastically reduced due to the lower availability of soluble protein C and the higher fraction of soluble thrombomodulin in the pulmonary edema fluid of these patients [5]. Therefore, anticoagulant therapy with inhaled APC might restore the natural anticoagulant cascades in the alveolar compartment of ALI/ARDS patients. Recent studies in several animal models of ALI have found that nebulized administration of APC attenuates lung injury [12]–[14] and reduces pulmonary coagulopathy without systemic anticoagulant effects [15]. However, little is known about the mechanisms through which APC could affect alveolar barrier integrity. APC attenuated thrombin-induced extracellular-regulated kinase 1/2 (ERK) activation in alveolar epithelial cells, a pathway involved in endothelial cell contraction and barrier dysfunction [16]. Nevertheless, to date, the direct role of APC in alveolar epithelial cell mechanical tension and barrier integrity in response to thrombin has not been investigated.

We aimed to investigate the effects of APC on the stiffening, contractility, and barrier integrity of human alveolar epithelial cells subjected to thrombin.

## Methods

### Reagents and Antibodies

Unless otherwise specified, reagents were obtained from Sigma (St. Louis, MO, USA). APC (Drotrecogin Alfa [activated]) was obtained from Eli Lilly and Company (Indianapolis, IN, USA).

### Cell Culture

The study was carried out on human lung epithelial cells: A549, culture line CCL-185; H441, culture line HTB-174 (American Type Culture Collection; Manassas, VA, USA); and primary culture of human alveolar epithelial cells (HAECs) isolated from human lung tissue (Innoprot; Bizkaia, Spain). HAECs comprise alveolar type I and type II epithelial cells. A549 and H441 cells were cultured in HEPES-buffered RPMI 1640 medium (GIBCO; Gaithersburg, MD, USA) supplemented with 10% inactivated fetal calf serum (GIBCO), 1 mM L-glutamine, penicillin–streptomycin (50 U/ml, 0.05 mg/ml, respectively), and 2.5 µg/ml amphotericin B. HAECs were propagated in the manufacturer’s recommended alveolar epithelial cell medium with 2% fetal bovine serum.

### Experimental Protocols

Cells grown to confluence were pretreated with 50 µg/ml APC (experimental cells) or culture medium (vehicle) for 3 h. Subsequently, 50 nM thrombin or culture medium was added to the cell culture. Cells pretreated with vehicle for 3 h and challenged with culture medium instead of thrombin were defined as control group. We conducted preliminary experiments with A549 cells to determine the dose of APC necessary to reduce thrombin-induced cell stiffening (0.1 µg/ml, 2.5 µg/ml, 5 µg/ml, or 50 µg/ml) and to evaluate whether the duration of APC pretreatment (1 h or 3 h) affected the reduction of cell stiffening (Figure S2).

### Cytotoxicity of APC Against A549 Cells

The cytotoxicity of APC, at 0.1 µg/ml, 2.5 µg/ml, 5 µg/ml, and 50 µg/ml, against A549 cells was evaluated after 72 h of APC stimulation by MTT Cell Proliferation Assay (R&D systems; Minneapolis, MN, USA) (Figure S1). This colorimetric assay measures the activity of cellular enzymes that reduce the tetrazolium dye, MTT, to its insoluble formazan, reflecting the cell proliferation rate and conversely, when metabolic events lead to apoptosis or necrosis, the reduction in cell viability.

### Measurement of Monolayer Permeability

The dynamics of barrier function were monitored using a real-time cell analyzer (RTCA SP) (xCELLigence System, Roche Applied Science; Mannheim, Germany). This system measures electrical impedance across the cell monolayer, cell impedance, by means of gold microelectrodes integrated on the bottom of tissue culture E-plates (Roche Applied Science; Mannheim, Germany). The E-Plate 96 has the same layout as conventional 96-well culture plates, but each individual well on an E-Plate incorporates a circle-on-line sensor electrode array. Therefore, approximately 80% of the area on the bottom of an E-Plate 96 is covered with gold microelectrodes. The device station, which is connected with the E-plate, is placed in the incubator and connected to the electronic RTCA analyzer through electrical cables. Cell impedance (CI) is a frequency-dependent parameter derived from impedance change according to CI = (Z_i_ – Z_0_)/15 Ω, where Z_i_ is the impedance at time i (i = 1, 2, …, N) and Z_0_ is the impedance at time 0. CI was measured at 10 kHz. When there are no cells in the wells, Z_i_ = Z_0_, and therefore CI = 0. A total of 2×10^4^ A549 cells or 5×10^4^ HAECs/H441 cells were cultured in each well. The E-plate 96 was placed in the incubator for at least 30 min before starting the experiment to ensure that cells were settled in the bottom of the well and was then inserted into the device station. Cell proliferation was assessed for 48 h, at which time the cells reached a sustained maximum CI value. At this time, cells were treated in tetraplicate as described above and CI was measured every minute up to 70 minutes after thrombin challenge. For each treatment, 7 experiments were carried out. In a series of experiments, A549 cells were first pretreated with RCR- 252 (20 µg/ml), an EPCR-blocking antibody that inhibits APC binding, or vehicle (culture medium) for 30 minutes before APC (50 µg/ml) exposure and 5 minutes of thrombin challenge.

### Measurement of Cell Stiffness

Cell stiffness was assessed by optical magnetic twisting cytometry (OMTC) [17]. Briefly, A549 cells were grown to confluence and pretreated with APC (50 µg/ml) or culture medium as described above; 10 µg ferrimagnetic beads coated with a synthetic RGD (Arg-Gly-Asp) were added to the cell culture and allowed to bind tightly to the cytoskeleton through cell surface receptors at 37°C and 5% CO_2_. Then, the cells were washed with HEPES-buffered RPMI-1640 to remove unbound beads and the well was mounted on the OMTC experimental setup. The beads were permanently magnetized in the horizontal direction (150 mT, 10 ms) and sinusoidally twisted in the vertical direction (3 mT, 0.1 Hz). Cell stiffness was measured from the twisting torque and bead displacement. Thrombin or culture medium was then added to the cell culture after 1 minute of OMTC baseline recording, and cell stiffness was measured for 5 additional minutes. The baseline value of cell stiffness was computed as the average of data obtained 30s before adding thrombin or culture medium. Cell stiffness after thrombin challenge was computed as the average of data obtained between 4.5 and 5 min. This technique has been described in detail elsewhere [8], [18]. For each treatment, 10 wells were tested.

### Measurement of Cell Contraction

Cell contraction force was determined using traction microscopy (TM), as described elsewhere [9], [18]. Briefly, A549 cells were grown on a thin collagen-coated polyacrylamide gel disk with embedded fluorescent beads. These flexible substrates were prepared beforehand using a mixture of 0.2 µm fluorescent latex beads (Molecular Probes, Invitrogen Corporation; Eugene, OR, USA) with 2% acrylamide and 0.3% bis-acrylamide solution (Bio-Rad; Hercules, CA, USA). Gel disks (Young’s modulus, 365 Pa) prepared with this solution were attached to a coverslip and subsequently coated with 3 µg/cm^2^ rat tail collagen I. Twenty-four hours after plating, cells were pretreated with APC (50 µg/ml) or culture medium as described above. The sample with the cell culture was then placed on an inverted microscope (Eclipse TE 2000-E, Nikon; Melville, NY, USA) with a cooled charge-coupled device camera (Orca AG, Hamamatsu Photonics) to measure cell contraction. First, a bright field image of an isolated cell was captured to determine its boundary. Subsequently, the apical surface of the gel was focused, and a fluorescence image of the beads from the apical surface of the gel was acquired. Thrombin or culture medium was added to the cell culture and another fluorescent image was acquired 5 min after the treatment challenge. At the end of the measurement, cells were removed from the gel disk by brief exposure to trypsin. Finally, an additional fluorescent image was recorded to determine the position of the beads in the unstrained gel (reference image). The displacement field between each fluorescence image and the reference image was measured. The traction field was calculated from Young’s modulus of the gel and the displacement field. For each traction field, the total force magnitude was computed by integrating the magnitude of the traction field over the projected area of the cell. For each treatment, 15 wells were tested.

### Protein Extraction and Fractionation

A549 cells seeded in D100 dishes and grown to confluence were pretreated with 50 µg/ml APC (experimental cells) or culture medium (vehicle) for 3 h. Subsequently, 50 nM thrombin or culture medium was added to the cell culture. Five minutes later, cells were rinsed twice with phosphate-buffered saline (PBS) and incubated on ice for 30 minutes in the presence of 500 µl of extraction buffer (20 mM Tris-HCl pH 7.4, 125 mM sucrose, 50 mM NaCl, 2 mM EGTA, 1 mM PMSF, 2 µl protease inhibitor, and 2 µl phosphatase inhibitor) for lysis. Cells were scraped and aspirated repeatedly through a 25-gauge needle. Then, cells were sonicated and ultracentrifuged at 100000×g for 30 minutes, with the cytosolic fraction being the supernatant product and the membrane/cytoskeleton fraction being the pellet product. The pellet was resuspended in 100 µl of extraction buffer. Protein concentration was determined using the bicinchoninic acid method (Pierce, Thermo Scientific; Rockford, IL, USA).

### Western Blot of the ZO-1 Protein

Equal amounts of protein were heat-denatured in sample-loading buffer (50 mM Tris-HCl, pH 6.8, 100 mM DTT, 2% SDS, 0.1% bromophenol blue, 10% glycerol) resolved in a 6% SDS-PAGE gel and transferred to PVDF membranes (GE Healthcare; Buckinghamshire, UK). Next, the blots were blocked with 5% PBS-non-fat dry milk for 2 hours at room temperature and then incubated with monoclonal mouse anti–ZO-1 (1.5 µg/ml) overnight at 4°C. Donkey anti-mouse peroxidase-conjugated IgG (Jackson Immuno Research; Suffolk, UK) was used as a secondary antibody, and α-tubulin antibody (Cell Signaling; Boston, MA, USA) was used as a loading control. Finally, 7 membrane-bound immune complexes were detected by an enhanced chemiluminescence system (ECL) (GE Healthcare) and the images were visualized and analyzed using an LAS3000 system (Fujifilm Life Science; Woodbridge, CT, USA).

### Immunofluorescence Microscopy

A549 cells grown to confluence on coverslips were pretreated with 50 µg/ml APC (experimental cells) or culture medium (vehicle) for 3 h. Subsequently, 50 nM thrombin or culture medium was added to the cell culture. Five minutes later, cells were washed twice with PBS and fixed in 3.7% formaldehyde in PBS for 10 minutes at room temperature. After three washes with PBS, cells were permeabilized with a solution of 0.1% Triton X-100 in PBS for 3 min and stained with Texas-Red C_2_-maleimide (Tx-Red) (Molecular Probes, Invitrogen Corporation) diluted 1∶100 in PBS for 30 minutes. After three washes with PBS, cells were blocked with a solution of 2% bovine serum albumin in 0.1% Tris-buffered saline (TBS)–Tween 20 (TBS-T) (blocking solution) for 30 minutes and incubated overnight at 4°C with monoclonal mouse anti-ZO-1 (15 µg/ml) (Zymed laboratories, Invitrogen Corporation; Carlsbad, CA, USA). The following day, cells were washed three times with TBS-T, incubated with Alexa 488-conjugated goat anti-mouse IgG (Molecular Probes, Invitrogen Corporation) (1∶200), and kept in the dark at room temperature for 1 h. After three additional washes with TBS-T, cellular nuclei were stained with Hoechst 33342 (Molecular Probes, Invitrogen Corporation) (1∶5000) for 5 min at room temperature. Immediately, coverslips were washed three times with PBS, dried, and mounted on a microscopy slide with the mounting medium Mowiol (Calbiochem, Merck; La Jolla, CA, USA). Images were acquired using a confocal laser scanning microscope (Eclipse TE 2000-E, Nikon; Melville, NY, USA) with a motorized XY stage MS-2000 (ASI; Eugene, OR, USA) and a 60× oil immersion objective; images were processed with MetaMorph 7.1 Software (Molecular Devices; Sunnyvale, CA, USA) to analyze the length of tight junction ZO-1 aggregates. A total of 27 images per treatment were used to quantify the ZO-1 length between cells.

### Statistical Analysis

Data are reported as mean ± SE. Comparisons between results obtained from different experiments were carried out by one-way ANOVA followed by the post-hoc Student-Newman-Keuls test, with the exception of APC dose-response and time-dependence results, which were assessed by unpaired Student’s t-test. When data failed the normality test in the one-way ANOVA, the Kruskal–Wallis one-way ANOVA on ranks was used. Statistical significance was set at p<0.05.

## Results

### Changes in Human Lung Epithelial Permeability Induced by Thrombin and APC

**Figure 1:**
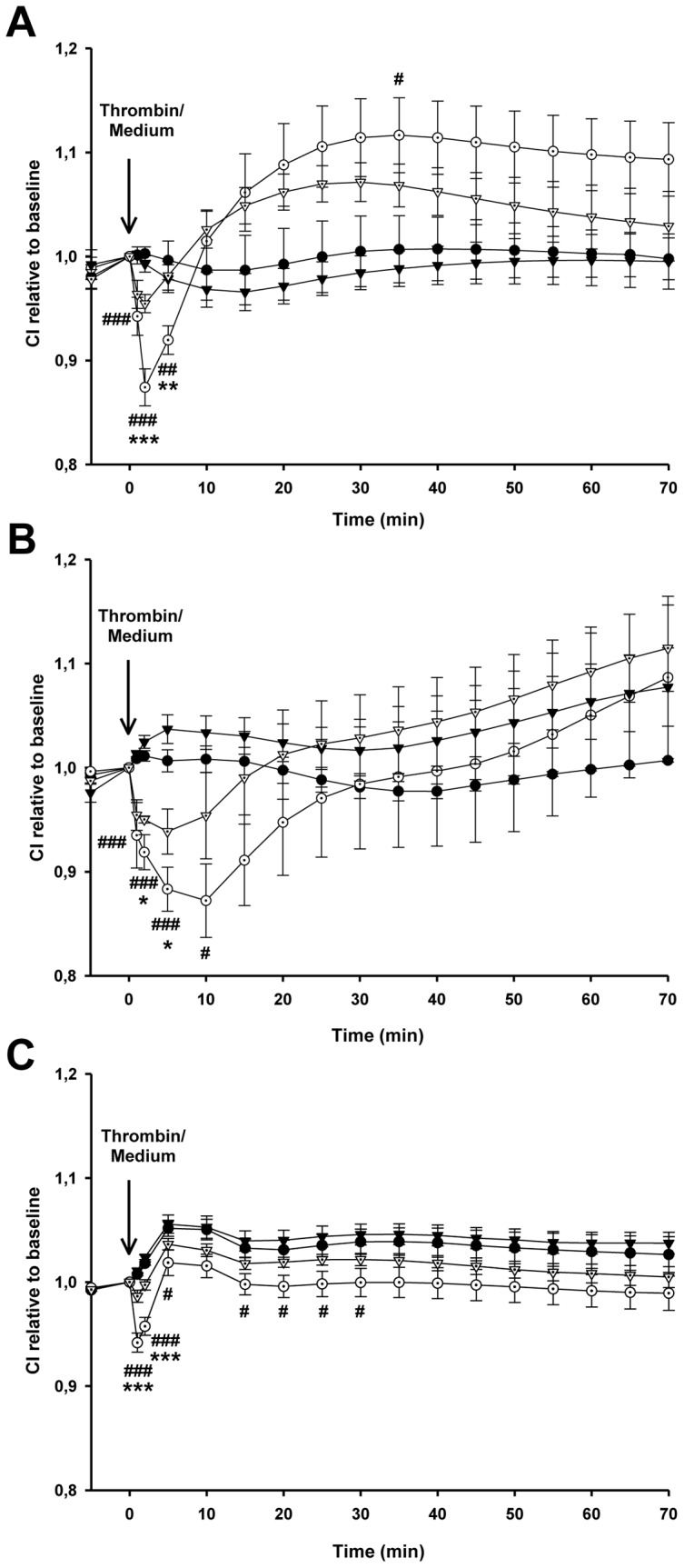
Effect of APC pretreatment on cell permeability of human lung epithelial monolayers exposed to thrombin. Cellular impedance (CI) was measured through electrical impedance across the human lung epithelial monolayers. At the time that confluent human lung adenocarcinoma cell lines A549 (A) and H441(C) and primary culture of human alveolar epithelial cells (HAECs) (B) reached a sustained maximum CI value, cells were pretreated with APC (50 µg/ml) or culture medium (vehicle) for three hours. Subsequently, thrombin (50 nM) or culture medium was added to the wells and CI was measured every minute up to 70 minutes after thrombin challenge. Black circles represent control cells (pretreated with vehicle for 3 h and then challenged with culture medium); white circles represent cells pretreated with vehicle and exposed to thrombin (50 nM); black triangles represent cells pretreated with APC (50 µg/ml) and challenged with culture medium; and white triangles represent cells pretreated with APC (50 µg/ml) and exposed to thrombin (50 nM). Data are reported as mean ± SEM. * and # denote significant differences between the vehicle + thrombin and APC + thrombin groups and between the vehicle + thrombin and control groups, respectively. Statistical significance was set at P<0.05. Measurements of monolayer permeability were performed on seven wells for each condition.

The acute response to thrombin produced a significant decline in CI in all three cell types: 12.6% in A549 (Figure 1A), 13.5% in HAECs (Figure 1B), and 5.8% in H441 (Figure 1C), reflecting increased permeability. This decline in CI was rapid, reaching a low point between 1 and 10 minutes after thrombin challenge. Thereafter, the pattern of the response to thrombin in the three human lung epithelial cell types differed. In A549 cells, CI increased rapidly for 20 minutes after reaching the low point and remained high for up to 70 minutes. In HAECs, CI increased slowly and progressively. In H441 cells, CI immediately returned to baseline values but remained lower in comparison to the control group throughout the 70 minutes. The thrombin-induced acute decline in CI was significantly attenuated by pretreatment with APC in all three human lung epithelial cell types (4.6% in A549 cells, 5.9% in HAECs, and 1.4% in H441 cells). This indicates that APC prevents the thrombin-induced increase in human lung epithelial permeability. No significant differences in CI values were found between cells pretreated with APC alone and control cells.

As the acute response to thrombin showed similar behavior in all three human lung epithelial cells, all further experiments were carried out in A549 cells and at 5 minutes.

### Effect of APC on Mechanical Tension of A549 Monolayers Exposed to Thrombin

**Figure 2:**
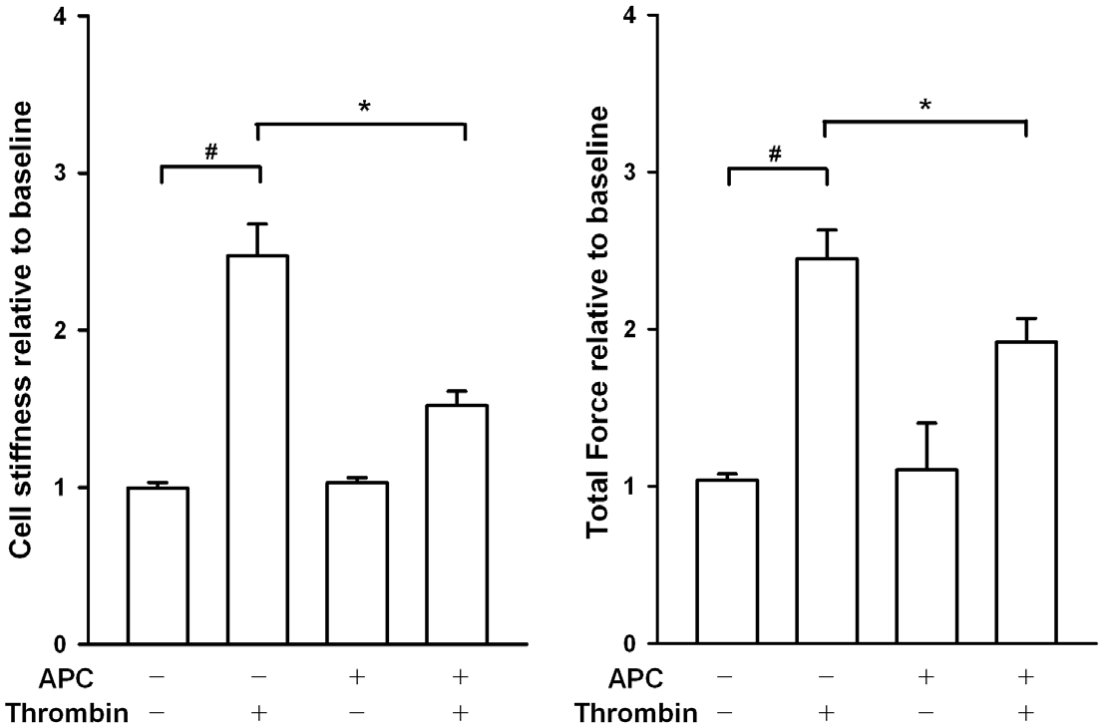
Effect of APC pretreatment (50 µg/ml) on cell mechanics in response to thrombin. Confluent A549 cells were pretreated with APC (experimental cells) or culture medium (vehicle cells). Cell stiffness and normalized total force magnitude was measured before (baseline recording) and 5 minutes after thrombin or culture medium challenge. Values are normalized to baseline values and reported as mean ± SEM. The statistical significance of the results was assessed by one-way ANOVA. * and # indicate significant differences between the vehicle + thrombin and APC + thrombin groups and between the vehicle + thrombin and control groups, respectively (p<0.05).

Figure 2 shows that thrombin induced a 2.5-fold increase in cell stiffness (*vs*. baseline data); pretreatment with APC significantly attenuated the increase to 1.5-fold. Thrombin induced a 2.5-fold increase in cell contraction (total force *vs*. baseline data); pretreatment with APC significantly attenuated the increase to 2.0-fold. No significant changes were found between APC-pretreated and control cells in either measurement of cell mechanics.

### Role of ZO-1 in APC- and Thrombin-mediated Dynamics of Alveolar Epithelial Barrier Integrity

**Figure 3:**
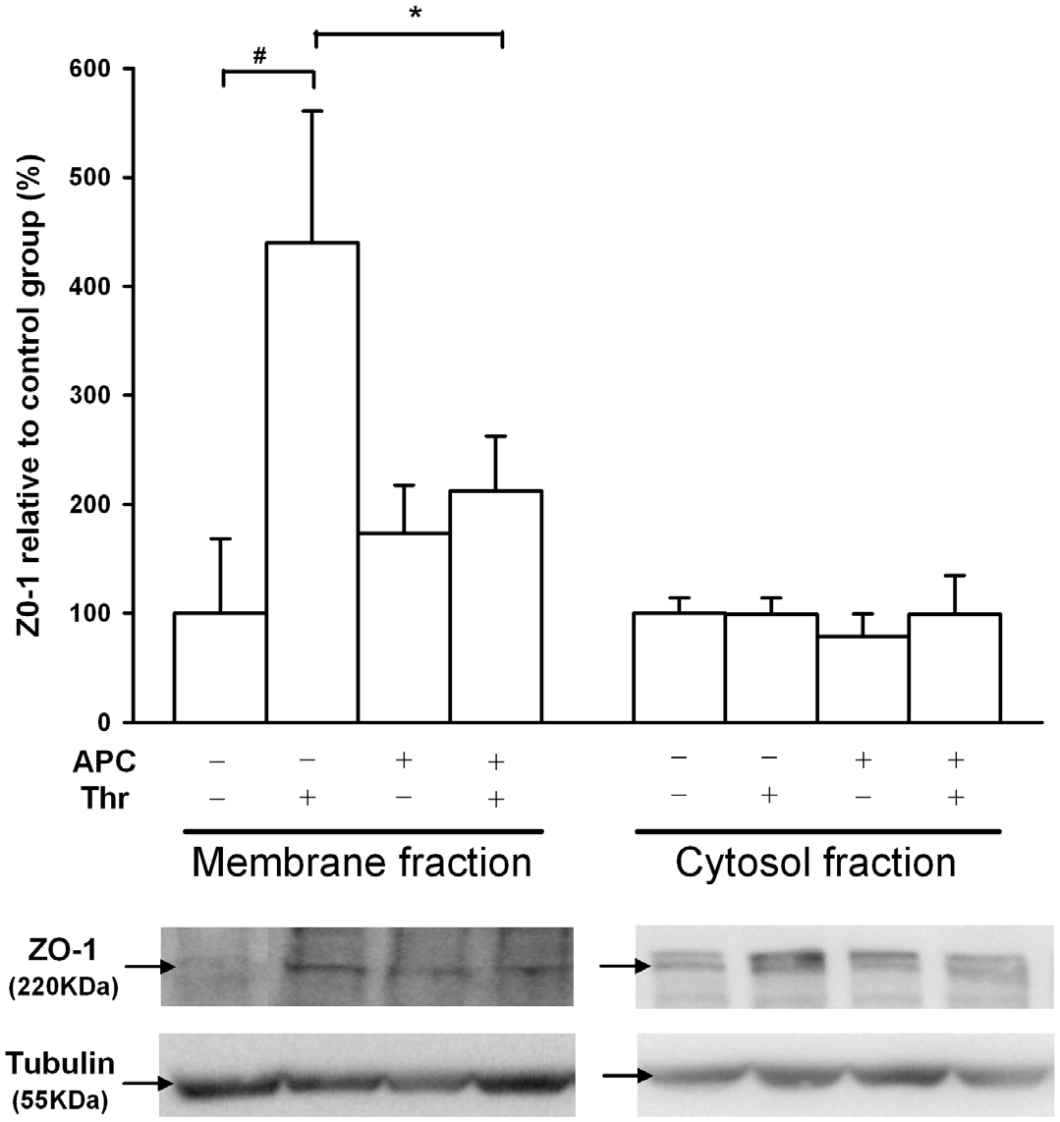
Recruitment of the tight junction protein ZO-1 at cell membrane after APC and thrombin exposure. Levels of ZO-1 protein in the membrane/cytoskeleton and cytosolic fractions were evaluated by western blot. A549 cells were pretreated with APC at 50 µg/ml or vehicle for 3 h, followed by thrombin (50 nM) or culture medium treatment for 5 min. Tubulin was used as an invariant control. Data from seven independent experiments are reported as mean values ± SEM. The results are expressed as a percentage of control (vehicle + culture medium group). The statistical significance of the results was assessed by one-way ANOVA (p = 0.038). * and # indicate significant differences between the vehicle + thrombin and APC + thrombin groups and between the vehicle + thrombin and control groups, respectively (p<0.05).

Cytosolic and membrane/cytoskeletal fractions were isolated from A549 cells and subjected to western blotting using ZO-1 antibody. Thrombin induced a 4.4-fold increase in ZO-1 membrane protein levels (percentage compared to control group) (Figure 3) that was significantly reduced (p<0.05) to a 2.1-fold increase when cells were pretreated with APC. Pretreatment with APC alone did not significantly modify the membrane protein levels compared to the control group. In addition, no statistically significant differences were observed in the cytosolic fraction in any of the conditions evaluated.

**Figure 4:**
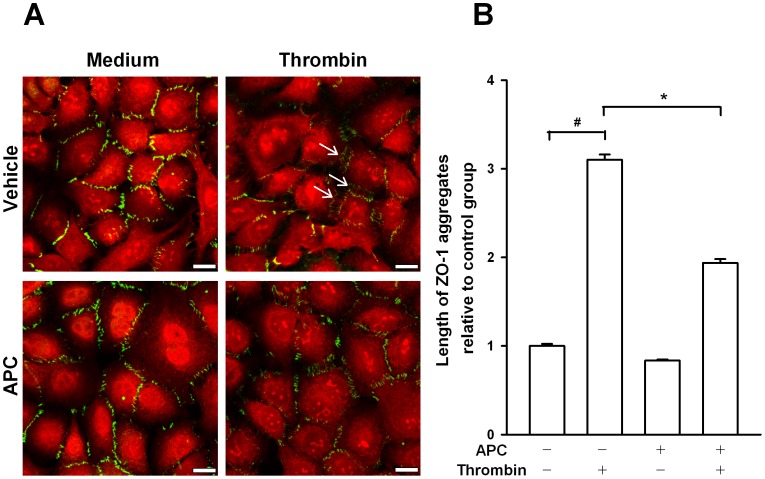
Effect of APC pretreatment (50 µg/ml) on tight junction ZO-1 integrity after thrombin challenge. The location and length of the tight junction ZO-1 in alveolar epithelial cells was assessed by immunofluorescence. A549 cells were pretreated with APC at 50 µg/ml or vehicle for 3 h. Subsequently, thrombin or culture medium was added to the cell culture for 5 min. In Figure A, ZO-1 fluorescence is indicated by green and Texas-red maleimide staining, which shows cell membranes, in red. Arrows show elongated aggregates of ZO-1 at cell-cell contacts. Representative results of 3 independent experiments. Scale bar = 10 µm. Figure B shows the quantification of ZO-1 length at cell-cell contacts of A549 cells; 27 images per treatment were used to quantify the ZO-1 length between cells. Results are reported as mean ± SEM. * and # denote significant differences between the vehicle + thrombin and APC + thrombin groups and between the vehicle + thrombin and control groups, respectively (p<0.05).

On immunofluorescence images, we limited the observation of ZO-1 to the areas where cells were in contact, because A549 cells are unable to create a tight monolayer (Figure 4A). In control conditions, ZO-1 appeared as discontinuous aggregates at cell-cell contacts, and the appearance of ZO-1 staining in cells pretreated with APC was similar. When cells were challenged with thrombin (50 nM), these ZO-1 aggregates become more elongated and assembled perpendicular to cell-cell interface areas (Figure 4, arrows). These elongated aggregates induced by thrombin seemed to be attenuated in cells pretreated with APC. The length of this tight junction was quantified using immunofluorescent images (Figure 4B). Thrombin induced a 3.1-fold increase in ZO-1 aggregate length (relative to the control group); pretreatment with APC for 3 h significantly reduced the thrombin-induced increase in ZO-1 aggregate length to 1.9-fold.

## Discussion

In the present study, we measured the effect of APC on cell stiffness, cell contraction, and cell barrier integrity in human alveolar epithelial cells subjected to thrombin. The collective findings show that APC pretreatment significantly reduced the effects of thrombin on cell mechanics and cell barrier integrity in the A549 monolayer, suggesting a barrier-protective response.

The structural integrity of the alveolar epithelial monolayer is regulated by the balance between inward tension forces and outward adhesive tethering forces at cell-cell and cell-matrix contacts. In this simple model, paracellular gaps form and the barrier is disrupted when adhesive cell-cell and cell-matrix tethering forces that regulate cell shape are unable to withstand cell tension forces exerted by cytoskeleton viscoelastic recoil and contraction of the actomyosin machinery [3]. In particular, thrombin can modulate this force balance [8]–[10], [18], acting near the site of its generation [19] through protease-activated receptors (PAR-1, PAR-3, and PAR-4).

We found that thrombin increased permeability in human lung epithelial cells (Figure 1) during the acute response. Although the establishment of functional tight junctions and the formation of impermeable monolayers by A549 cells has been questioned [20], [21], the acute thrombin response in A549 cell permeability was similar to that of HAECs composed of alveolar type I and type II epithelial cells and of H441 cells, a respiratory epithelial cell line that is able to form confluent monolayers. Based on these findings and the fact that A549 cells can also express different proteins involved in the coagulation cascade [5], [22], [23] and can generate new APC in the presence of thrombin and protein C antigen [24], we considered the A549 cell line an appropriate model to study the effects of APC on the structural integrity of the alveolar epithelial monolayer subjected to thrombin. Moreover, the acute thrombin response found in A549 cell permeability is in line with several data showing that thrombin induces a more marked effect after five minutes of treatment [8], [10], [14], [18]. In agreement with previous studies, we found that thrombin stiffens and contracts alveolar epithelial cells, suggesting an increase in inward forces compromising the barrier integrity [8], [9], [18]. In this study, we found that the thrombin-induced increase in cell stiffness and contraction is associated with a significant increase in cell permeability. Although some evidence suggests that thrombin compromises barrier integrity, other evidence suggests that thrombin can enhance cell adhesion in A549 cells, which could reinforce barrier integrity. In particular, thrombin induces the formation of actin bundles [8]–[10] and accumulation of MLC diphosphorylation in the peripheral ring [10]; and as previously reported [10], we also found cell membrane recruitment of the tight junction protein ZO-1.

The apparent divergence in the role of thrombin in alveolar epithelial cells could be explained by cell intrinsic mechanisms preserving the cell barrier integrity in response to an increase in centripetal tension. Recent data demonstrate that under the application of a local cell force, such as cell contraction inducing paracellular permeability, more adhesive proteins are recruited to the affected contacts to reinforce them [25], [26]. Previous studies reported that ZO-1 protein undergoes posttranslational modifications induced by different stimuli, such as phosphorylation [27], [28]. As many proteins belonging to the tight junctional complex, including ZO-1, are phosphoproteins [27], the rapid enhancement of these proteins in the cell membrane compartment is a consequence of changes in their phosphorylation state [28]. In fact, these phosphorylated changes are associated with tight junction functions and in particular with paracellular permeability [27]–[29]. Considering that thrombin induced an increase in A549 cell permeability, the thrombin-induced increase of ZO-1 protein levels in the membrane compartment could be the result of a posttranslational modification such as phosphorylation. However, further studies are needed to elucidate this mechanism. Moreover, on immunofluorescence images, the thrombin-induced elongated ZO-1 aggregates were situated perpendicularly in the areas where cells are in contact. This ZO-1 pattern was also observed in human pulmonary artery endothelial cells treated with thrombin [10] and is also very similar to that induced by some cadherins in different cell types [10], [30]–[32]. Brevier and coworkers [30] recently correlated the formation and length of the adherens junction VE cadherin with cell contractility. The larger the contractile force, the greater the length of the cell-cell junction aggregates formed between adjacent cells [30]. Under this hypothesis, the cell-cell contact needs to be linked to the actin-myosin apparatus; ZO-1 is connected directly to the perijunctional actin ring [33] and interacts with myosin through cingulin, a cytoplasmatic protein belonging to the tight junctional complex [34]. Considering the similarity of the pattern observed in adherens junctions in response to contractile agents to those observed in tight junctions, cell contraction could affect the length of the tight junction ZO-1. This interpretation is supported by our finding that the length of ZO-1 aggregates at cell-cell contacts for all cell treatments showed a linear dependence with the corresponding total force magnitude (Figure S3).

The anti-inflammatory effects of APC at the endothelial level have been widely studied [35]–[38]. By contrast, the role of APC in the alveolar epithelium is poorly understood. We found no significant differences in cell stiffness between APC-pretreated alveolar epithelial cells and control cells, at any of the four doses studied (data not shown). Moreover, no significant changes in cell permeability were found between any of the three human lung epithelial cells pretreated with APC and control cells. Likewise, no significant changes in cell contraction or in the dynamics of ZO-1 were found between A549 cells pretreated with APC and control cells. These results suggest that APC alone does not mediate mechanical or biological responses that may compromise the integrity of the alveolar epithelial barrier. This finding is in line with recent data showing that APC induces a minimal response on the phosphorylation of ERK1/2 [14], a mitogen-activated protein kinase involved in endothelial cell permeability [16]. On the other hand, we found that APC reduced thrombin-induced responses in human lung epithelial cells, as occurs in the endothelium. Maniatis and colleagues recently found that APC attenuates the activation of ERK1/2 induced by thrombin in alveolar epithelial cells [14], suggesting that APC could reduce the stress fiber formation and cell permeability induced by thrombin in the alveolar epithelium, as occurs in the endothelium [16]. However, to date, the direct role of APC in reducing thrombin-induced cell contraction, cell stiffness, and cell permeability in alveolar epithelial cells has not been reported.

Pretreatment with APC not only significantly reduced thrombin-induced A549 cell stiffening, cell contraction, and cell permeability, but also decreased the levels of ZO-1 in the membrane protein content and the number of elongated ZO-1 aggregates that were assembled perpendicular to cell-cell interface areas. APC also reduced thrombin-induced cell permeability in HAECs and in H441 cells, with a significant difference achieved in HAECs during the first 5 minutes of thrombin challenge.

Taken together, these results suggest that when inward forces increase in response to a contractile stimulus, such as thrombin, a protective intrinsic cell mechanism to preserve barrier integrity is also activated. However, when this contractile stimulus is minimized, such by the action of APC, the protective intrinsic cell mechanism in response to centripetal tension may not be entirely necessary. Therefore, the barrier-protective response to a contractile stimulus could depend on the strength of this stimulus.

One of the mechanisms by which APC mediates the barrier protective response in endothelial cells is cleaving and activating PAR-1 in an EPCR-dependent fashion [35]. As A549 cells can express EPCR [22], we sought to evaluate the role of this receptor in APC-preserved barrier integrity in response to thrombin. We found no significant differences in CI between the cells pretreated with an EPCR-blocking antibody that inhibits APC binding and vehicle cells (Figure S4). These results indicate that APC is able to reduce thrombin-induced responses in alveolar epithelial cells without binding to EPCR, suggesting that other mechanisms could be involved. Further studies are needed to elucidate these complementary mechanisms.

In conclusion, our results demonstrate that pretreatment with APC significantly reduces the effects of thrombin in alveolar epithelial cells, suggesting a barrier-protective response. We hypothesize that, as a consequence of thrombin-induced increases in inward forces, outward tethering adhesive forces were also reinforced through a protective intrinsic cell mechanism to preserve barrier integrity, such as ZO-1 recruitment at the cell membrane. This reinforcement could be minimized by the protective effects of APC. Further research using other stimuli will help us to better understand the dynamics of mechanical forces at cell-cell attachments and the role of different tight junctions on alveolar-capillary barrier disruption in *in vitro* models of ALI. Overall, our findings provide new insights into the protective effects of APC on the alveolar epithelial barrier by restoring the coagulation pathways in the alveolar space of ALI patients.

## Supporting Information

Figure S1 **Viability of A549 cells treated with APC at four different concentrations.** Cell viability was assessed at four different concentrations (0.1 µg/ml, 2.5 µg/ml, 5 µg/ml, and 50 µg/ml) for 72 h by MTT assay. According to the manufacturer’s instructions, an APC dose may be cytotoxic for A549 cells when cell viability is less than 80%. Values are reported as mean ± SEM. APC dose-response and time-dependence results.
(TIF)

Figure S2 **APC dose-response and time-dependence on cell stiffness in response to thrombin.** Cell stiffness was measured by optical magnetic twisting cytometry (OMTC). Confluent A549 cells were pretreated with APC at different conditions or vehicle. Thrombin (50 nM) or culture medium was added to the wells after OMTC baseline recording, and 5 minutes later cell stiffness was measured. (A) Cells were pre-treated with APC at four different concentrations (0.1, 2.5, 5, and 50 µg/ml) or vehicle (culture medium) for 1 hour. (B) A549 cells were pre-treated with the highest APC concentration (50 µg/ml), close bars, or vehicle, open bars, for 1 or 3 hours. Data are reported as mean ± SEM (***, **, and * indicate P<0.001, P<0.01, and P<0.05, respectively). OMTC measurements were performed on nine wells for each condition.
(TIF)

Figure S3 **Relationship between the length of ZO-1 aggregates at cell-cell contacts and the traction forces.** The median ZO-1 aggregate length for each group is plotted versus the median total force magnitude in the same conditions. Black circles represent control cells (pretreated with vehicle for 3 h and afterwards challenged with culture medium), white circles represent cells pretreated with vehicle and exposed to thrombin (50 nM), black triangles represent cells pretreated with APC (50 µg/ml) and challenged with culture medium, and white triangles represent cells pretreated with APC (50 µg/ml) and exposed to thrombin (50 nM). The line resulting from a linear fit of the data has a slope obtained of 0.6398 with R^2^ = 0.967.
(TIF)

Figure S4 **Role of EPCR in APC-mediated barrier-protective response.** Cells were first pretreated with RCR-252 (20 µg/ml), an EPCR-blocking antibody that inhibits APC binding, or vehicle (culture medium) for 30 minutes before exposure to APC (50 µg/ml). After three hours, cells were challenged with thrombin (50 nM), and 5 minutes later cell impedance was measured. Cells were treated in tetraplicate. For each treatment, 6 experiments were carried out. Data are reported as mean ± SEM.
(TIF)
