# Supplementary figures and images for: Barrier-Protective Effects of Activated Protein C in Human Alveolar Epithelial Cells
Source: PLoS One. 2013 Feb 22;8(2):e56965. doi: 10.1371/journal.pone.0056965 (PMC3579945; doi:10.1371/journal.pone.0056965)

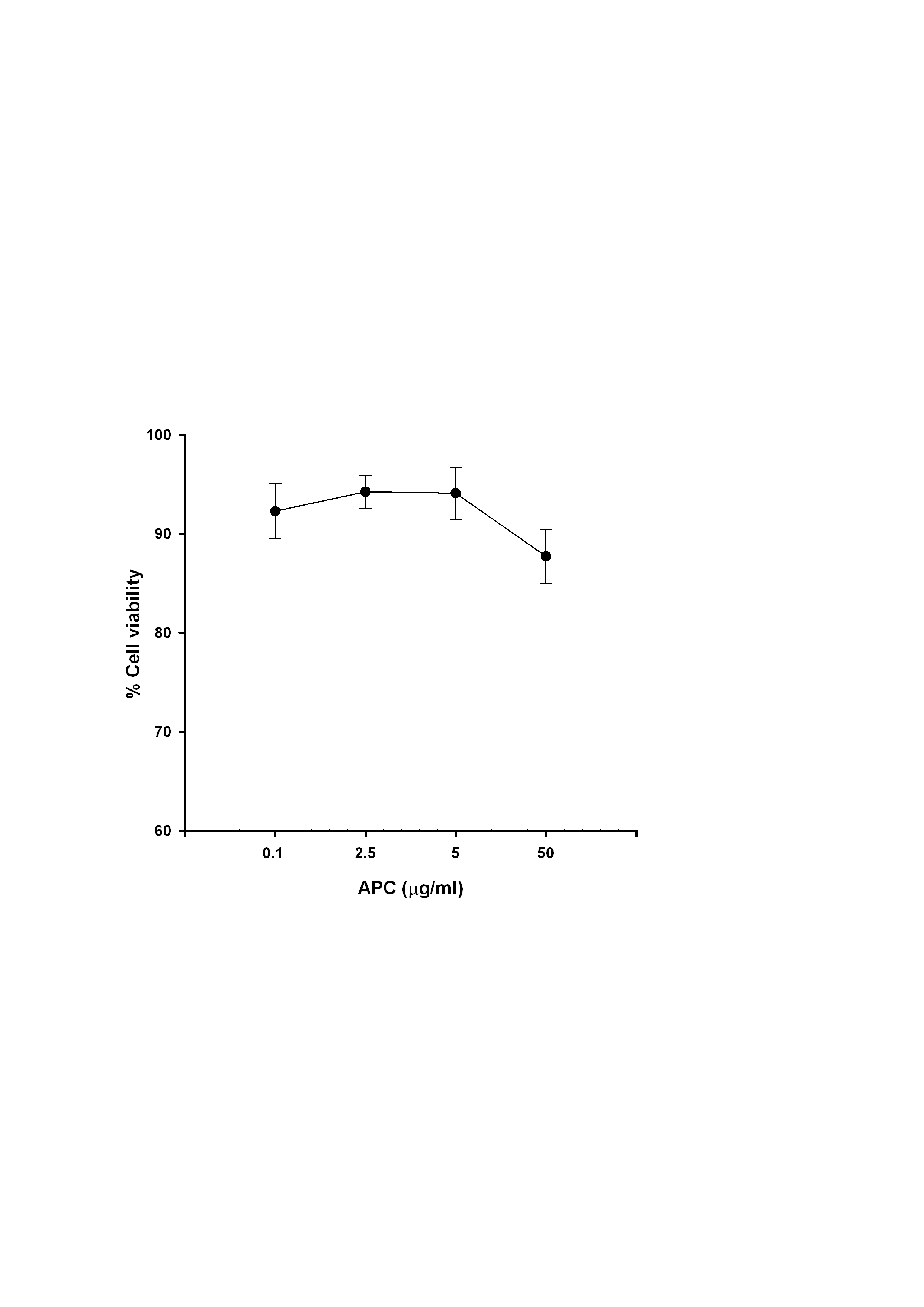

Supplement: Figure S1 — Viability of A549 cells treated with APC at four different concentrations. Cell viability was assessed at four different concentrations (0.1 µg/ml, 2.5 µg/ml, 5 µg/ml, and 50 µg/ml) for 72 h by MTT assay. According to the manufacturer’s instructions, an APC dose may be cytotoxic for A549 cells when cell viability is less than 80%. Values are reported as mean ± SEM. APC dose-response and time-dependence results. (TIF) [file pone.0056965.s001.tif]

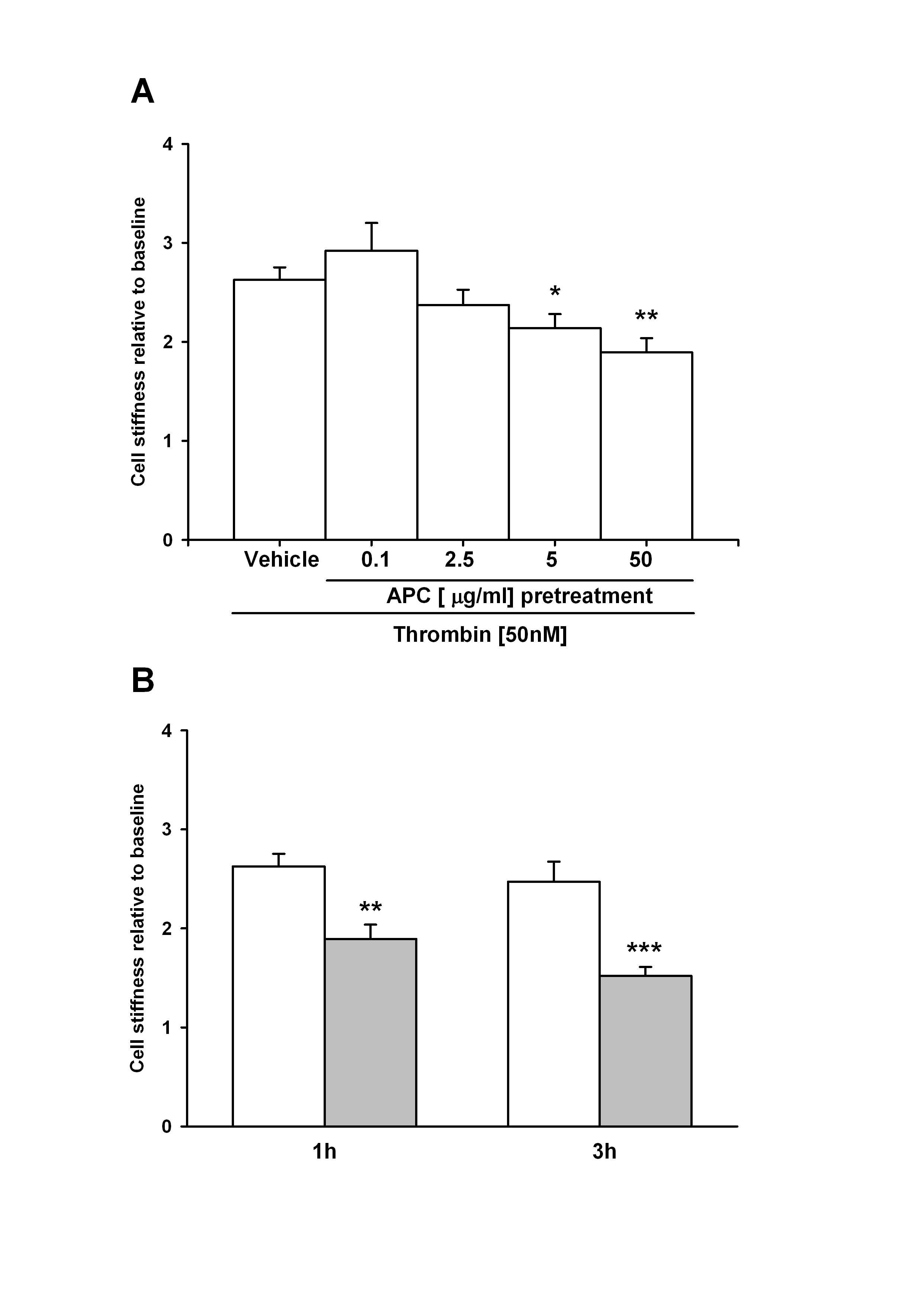

Supplement: Figure S2 — APC dose-response and time-dependence on cell stiffness in response to thrombin. Cell stiffness was measured by optical magnetic twisting cytometry (OMTC). Confluent A549 cells were pretreated with APC at different conditions or vehicle. Thrombin (50 nM) or culture medium was added to the wells after OMTC baseline recording, and 5 minutes later cell stiffness was measured. (A) Cells were pre-treated with APC at four different concentrations (0.1, 2.5, 5, and 50 µg/ml) or vehicle (culture medium) for 1 hour. (B) A549 cells were pre-treated with the highest APC concentration (50 µg/ml), close bars, or vehicle, open bars, for 1 or 3 hours. Data are reported as mean ± SEM (***, **, and * indicate P<0.001, P<0.01, and P<0.05, respectively). OMTC measurements were performed on nine wells for each condition. (TIF) [file pone.0056965.s002.tif]

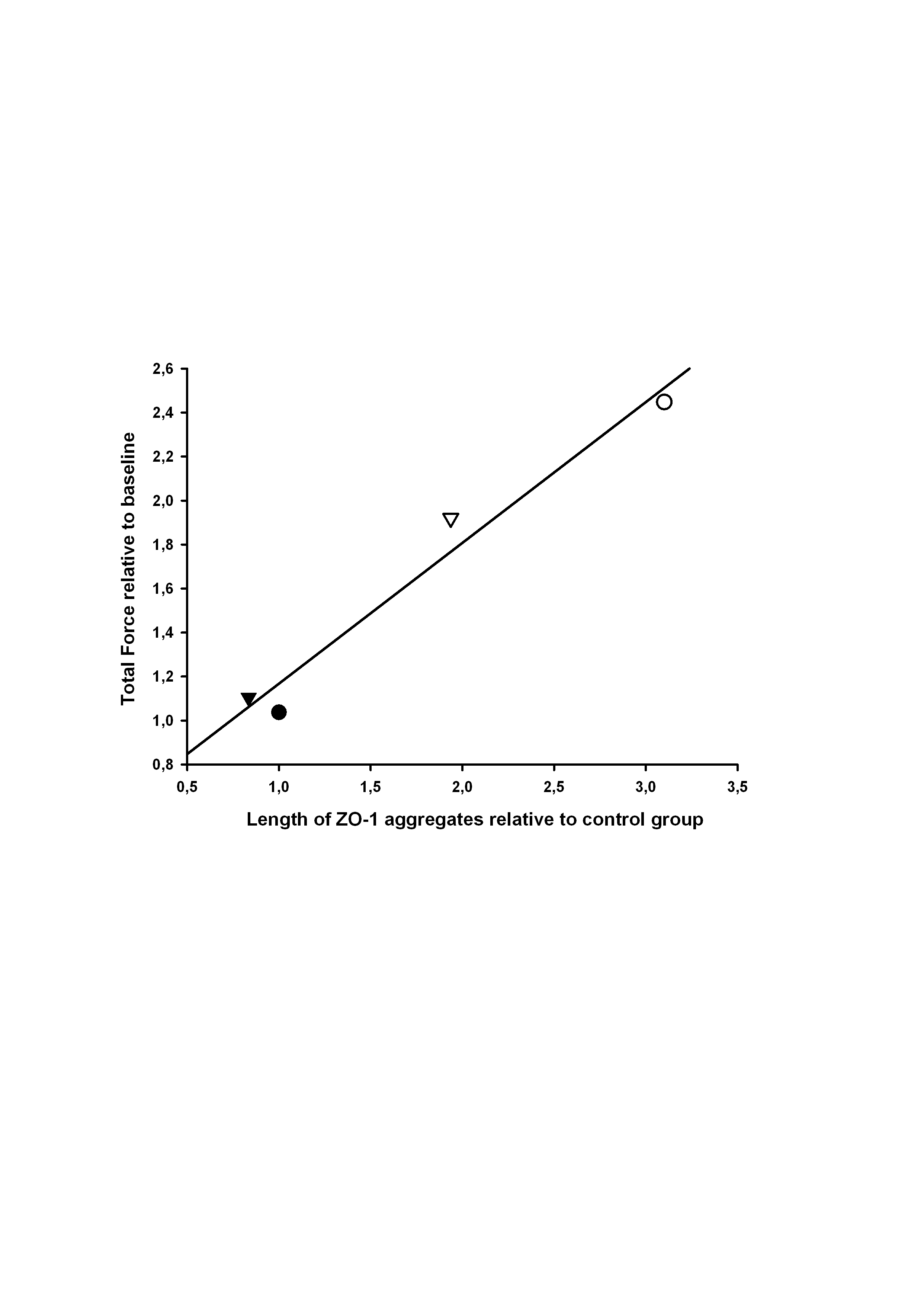

Supplement: Figure S3 — Relationship between the length of ZO-1 aggregates at cell-cell contacts and the traction forces. The median ZO-1 aggregate length for each group is plotted versus the median total force magnitude in the same conditions. Black circles represent control cells (pretreated with vehicle for 3 h and afterwards challenged with culture medium), white circles represent cells pretreated with vehicle and exposed to thrombin (50 nM), black triangles represent cells pretreated with APC (50 µg/ml) and challenged with culture medium, and white triangles represent cells pretreated with APC (50 µg/ml) and exposed to thrombin (50 nM). The line resulting from a linear fit of the data has a slope obtained of 0.6398 with R2 = 0.967. (TIF) [file pone.0056965.s003.tif]

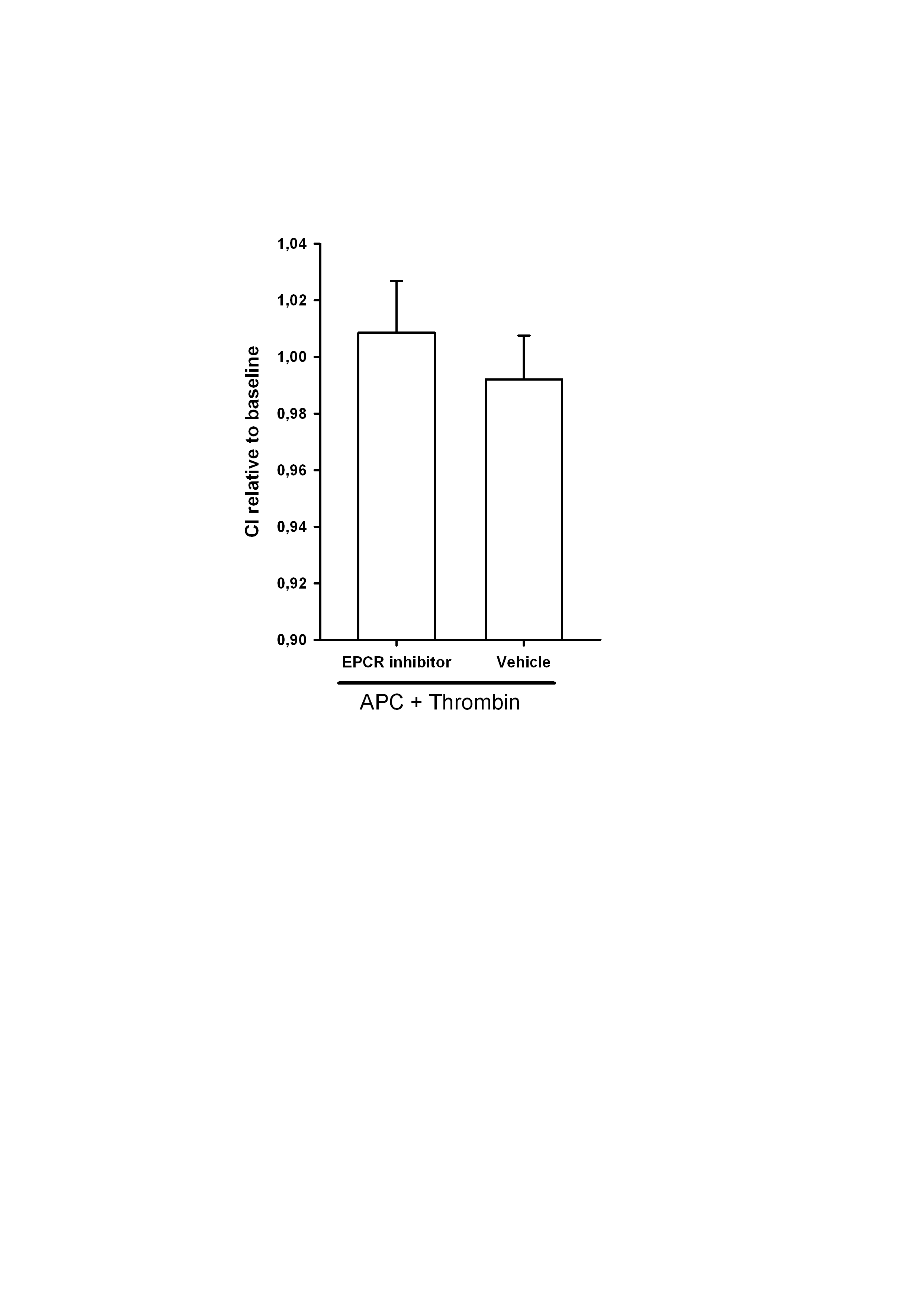

Supplement: Figure S4 — Role of EPCR in APC-mediated barrier-protective response. Cells were first pretreated with RCR-252 (20 µg/ml), an EPCR-blocking antibody that inhibits APC binding, or vehicle (culture medium) for 30 minutes before exposure to APC (50 µg/ml). After three hours, cells were challenged with thrombin (50 nM), and 5 minutes later cell impedance was measured. Cells were treated in tetraplicate. For each treatment, 6 experiments were carried out. Data are reported as mean ± SEM. (TIF) [file pone.0056965.s004.tif]
